# Three-Dimensional Multi-Agent Foraging Strategy Based on Local Interaction

**DOI:** 10.3390/s23198050

**Published:** 2023-09-23

**Authors:** Jonghoek Kim

**Affiliations:** System Engineering Department, Sejong University, Seoul 05006, Republic of Korea; jonghoek@gmail.com

**Keywords:** multi-agent foraging, 3D cluttered unknown workspace, foraging based on local interaction, provably complete search, multi-agent resource gathering

## Abstract

This paper considers a multi-agent foraging problem, where multiple autonomous agents find resources (called pucks) in a bounded workspace and carry the found resources to a designated location, called the base. This article considers the case where autonomous agents move in unknown 3-D workspace with many obstacles. This article describes 3-D multi-agent foraging based on local interaction, which does not rely on global localization of an agent. This paper proposes a 3-D foraging strategy which has the following two steps. The first step is to detect all pucks inside the 3-D cluttered unknown workspace, such that every puck in the workspace is detected in a provably complete manner. The next step is to generate a path from the base to every puck, followed by collecting every puck to the base. Since an agent cannot use global localization, each agent depends on local interaction to bring every puck to the base. In this article, every agent on a path to a puck is used for guiding an agent to reach the puck and to bring the puck to the base. To the best of our knowledge, this article is novel in letting multiple agents perform foraging and puck carrying in 3-D cluttered unknown workspace, while not relying on global localization of an agent. In addition, the proposed search strategy is provably complete in detecting all pucks in the 3-D cluttered bounded workspace. MATLAB simulations demonstrate the outperformance of the proposed multi-agent foraging strategy in 3-D cluttered workspace.

## 1. Introduction

This paper considers a multi-agent foraging problem in which multiple autonomous agents find resources (called pucks) in a bounded workspace and carry the found resources to a designated location called the base. Every agent has limited capabilities and lacks sophisticated hardware such as a Global Positioning System (GPS). Every agent executes simple decision procedures in a decentralized manner, and there is no leader.

Foraging has real-world applications, e.g., in the distributed cleanup of hazardous toxins or the automated construction and repair of artificial structures; in both of these situations, agents must first locate and then transport objects of interest (toxins in the former, building blocks in the latter) in a decentralized manner [1,2,3]. Collection and transport tasks have various applications, such as mine-clearing, hazardous waste cleanup, and search and rescue.

Our paper handles multi-agent foraging, which involves multiple agents collecting randomly distributed resources (pucks) and carrying them back to a single position (base). We address 3D multi-agent foraging based on local interaction, which does not rely on global localization of an agent. In other words, GPS is not utilized by the agents. In this problem, multiple agents begin moving from the base with the goal of gathering all of the pucks, which are randomly distributed in the workspace. In the proposed solution, each agent is controlled such that they carry the sensed pucks back to the base while moving based on local interaction with their neighbors. In this way, an agent’s maneuvering does not depend on global localization.

We assume that each agent has local sensing, local communication, and movement abilities. Each agent can detect a nearby puck using its local sensors, such as a camera. We consider a case in which the agents move in an unknown 3D workspace with many obstacles that are not known in advance. In this case, obstacles can block a communication link between agents and hinder the motion of each agent. Each agent lacks any global localization or global communication.

If an agent moves too far away from any other agent, it cannot communicate with the others due to the limited sense-communication range. Hence, an agent must stay within sense-communication range of at least one other agent while exploring the unknown 3D cluttered workspace.

Our study considers a bounded 3D workspace which contains many unknown obstacles. Our search strategy is provably complete in detecting all pucks in a 3D cluttered bounded workspace which is not known in advance. We prove that under the proposed search strategy all pucks in the unknown bounded workspace are found in finite time.

To the best of our knowledge, no paper in the literature has handled the 3D multi-agent foraging problem in our article. Moreover, our article is novel in letting multiple agents perform foraging and puck carrying in a 3D unknown cluttered workspace while not depending on global localization of an agent. We only use local interaction with maximum sense-communication range.

The proposed foraging strategy has the following two steps. The first step in the proposed foraging strategy is to detect every puck in the 3D cluttered unknown workspace in a provably complete manner. Initially, all agents are located at the base. The search process is performed by moving agents one by one to expand the sensor coverage, until no coverage holes remain in the 3D cluttered bounded workspace. The 3D cluttered workspace is considered completely covered when every puck has been sensed by at least one agent.

The second step in foraging is to collect the pucks and return them to the base. In the collection step, global localization and global communication cannot be utilized. Because the agents cannot use global localization, each agent moves based only on local interactions within its limited sense-communication range.

The novel contributions of this paper are summarized as follows:

1. This article is novel in allowing multiple agents to perform foraging and puck search in a cluttered 3D workspace while not relying on global localization of agents.
2. Our search strategy is provably complete in detecting all pucks in a 3D cluttered bounded workspace which is not known in advance.
3. Our paper is novel in addressing how to return all pucks to the base in such a way that global localization and global communication are not required.

MATLAB simulations are used to demonstrate the performance of our proposed 3D foraging strategy.

The remainder of this article is structured as follows: Section 2 presents a literature review of related papers; Section 3 describes the preliminary information of this article; Section 4 describes the definitions and assumptions in this paper; Section 5 describes the 3D multi-agent foraging strategy; Section 6 addresses the MATLAB simulations; finally, Section 7 presents our conclusions.

## 2. Literature Review

Area coverage methods using multiple agents can be applied to detect randomly distributed pucks in the workspace. In [4,5,6,7], the authors handled how to make multiple agents perform area coverage in a 2D workspace. In [8,9], the authors utilized 2D Voronoi tessellations for making agents expand the agent network based on information sharing with neighbors. However, Refs. [8,9] did not consider obstacles in the workspace. The authors of [10] considered multiple agents that are instructed to cover a field of interest (FoI). After agents complete a task at one FoI, they move to a new FoI, which may be far away from the current FoI. According to [10], agents can automatically adjust their deployment density in the new FoI based on the requirements of various tasks or regions.

However, coverage strategies for an 3D cluttered unknown workspace were not considered in these papers. Moreover, the authors did not handle multi-agent foraging, which involves multiple agents collecting randomly distributed pucks and carrying them back to the base.

There are many papers on multi-agent foraging strategies inspired by nature, e.g., ant, bee, and physarum polycephalum [2,3,11,12,13,14,15,16]. Inspired by nature, Refs. [17,18] showed that random searches relying on puck distribution can be optimized by considering individuals in foraging. The superdiffusive property of Levy walks, in which the mean squared displacement increases superlinearly [12,19], is a known mechanism that nature uses to handle low information levels from sensory systems. Inspired by the behaviour induced by pheromones released by ants, navigation methods based on landmarks or markers such as RFID tags were addressed in [15,20,21]. Even an extremely sensor-poor and processing-poor robot can perform navigation when accessing markers [21] or RFID tags [20]. Inspired by the pheromone trails of ants, Refs. [2,3,22,23] addressed robotic systems mimicking ants’ foraging. In [16], the author developed a virtual pheromone (VP) algorithm inspired by the foraging behavior of ants, with the exception that the agents themselves act as pheromone locations (beacons) and these virtual pheromones are laid and detected through direct local agent–agent communication. The authors of [14] proposed foraging algorithms inspired by bees. In [13], the foraging process (search and contraction) of physarum polycephalum, a unicellular and multi-headed slime mold, was simulated utilizing multi-agent systems.

However, mimicking natural behaviors cannot guarantee that every puck is detected by an agent within a finite time. In other words, mimicking natural behavior is not provably complete for searching all pucks. Moreover, it is not clear what hardware can perform the functionality of a pheromone. In our paper, we specify the sensor systems required for the proposed foraging strategy.

To cover a workspace with large uncertainty, random searches may be beneficial; however, random maneuvering can easily lead to a lost agent in cases where GPS is not available. For maintaining network connectivity, each agent needs to move while considering the maximum sense-communication range of an agent. This inspired us to develop a multi-agent foraging strategy which relies on local sensors with the maximum sense-communication range. In our paper, the proposed search strategy is provably complete in detecting all pucks in a 3D bounded workspace which is not known in advance.

Advances in reinforcement learning have recorded success in various domains. Considering a 2D small workspace with no obstacles, Ref. [24] formulated multi-robot learning for learning foraging behavior in a group of four ground robots. In [25], the author investigated the application of reinforcement learning as a rehearsal (RLaR) for the multi-agent foraging problem. However, the reinforcement learning approach in [25] cannot assure that every puck is found under the subsequent search strategy. The fundamental problem in multi-agent reinforcement learning is, as always, the curse of dimensionality [26,27]. The state–action space and the combinatorial possibilities of agent interactions increase exponentially with the number of agents, which renders sufficient exploration a difficult problem by itself. This is intensified when agents only have access to partial observations of the environment [27]. Therefore, open questions remain, such as how to sufficiently explore large and complex spaces and how to solve large combinatorial optimization problems [27].

In our paper, agents explore 3D cluttered environments which are not known in advance. Therefore, each agent only has access to local information, on which individual decisions must be taken. In other words, agents have access to only partial observations of the environment. In our paper, the proposed search strategy is provably complete in detecting all pucks in a 3D bounded workspace which is not known in advance. Our MATLAB simulation shows that the proposed foraging strategy runs quickly and is suitable for real-time applications.

## 3. Preliminary Information

### 3.1. Graph Theory

Utilizing graph theory [28], one can define a graph as G=(V(G),E(G)) such that V(G) is the node set and E(G) is the edge set. Two nodes of an edge e∈E(G) are called *neighbors*. A *path* is a sequence of connected edges. A graph *G* is considered *connected* if a path can be established between any two of its nodes.

A *cycle* is a graph building a closed chain. A *tree T* is a connected graph containing no cycles. A *spanning tree* of a graph *G* is a tree which contains all nodes in *G*.

One node of *T* is set as the *root*. For convenience, let *v* denote a node in *T* and let N(v) denote a *neighbor* of *v*, which is one hop distance from *v*. In *T*, p(v) (the *parent* of *v*) is the neighbor of *v* along the path to the root. Furthermore, c(v), the *child* of *v* is a node such that *v* is the parent of c(v).

**Figure 1:**
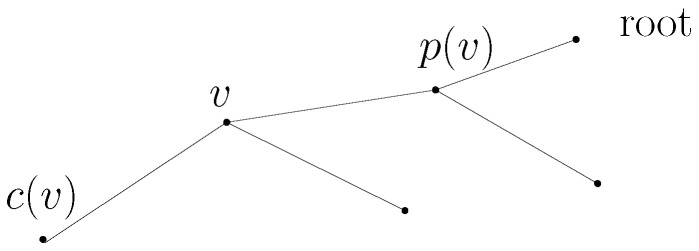
Tree illustration with c(v) and p(v).

A *leaf* is a node having no children. A *descendant* of *v* is a node which is either c(v) or the descendant of c(v) (recursively). An *ancestor* of *v* is a node which is either p(v) or the ancestor of p(v) (recursively). Figure 1 depicts a tree illustrating c(v) and p(v).

### 3.2. Problem Statement and Foraging Strategy Introduction

Our paper handles multi-agent foraging, which involves multiple agents collecting randomly distributed pucks and carrying them back to a base. To make the problem more interesting, in this article we solve the problem of 3D multi-agent foraging in a GPS-denied bounded workspace with many unknown obstacles. The goal of our multi-agent foraging problem is as follows. *Considering an unknown 3D cluttered bounded workspace, multiple agents begin moving from the base to search for all of the randomly distributed pucks. Each agent is controlled such that they carry the sensed pucks back to the base. All agents must move based only on local interaction with their neighbors, without relying on global localization.*

The proposed foraging strategy has the following two steps. The first step is to detect every puck in the 3D cluttered bounded workspace in a provably complete manner. Initially, all agents are located at the base. As an exceptional situation, we further handle the case where a number of agents are not initially located at the base (Section 5.1.3).

The search process is performed by moving agents one by one to expand the sensing coverage until no coverage holes remain. The 3D cluttered workspace is considered to have been completely covered when every puck has been sensed by at least one agent. This provably complete puck search is presented in Section 5.1.

The second step is to return every puck to the base. In the collection step, no global localization or global communication is utilized. This collection step based on local interaction is presented in Section 5.2.

## 4. Assumptions and Notation

We consider discrete-time systems. Let dt define the sampling interval in a discrete-time system. For instance, in the case where our controls are applied at every 2 seconds, dt=2 s.

Consider the case where the 3D workspace is closed and bounded. There are unknown obstacles inside the 3D workspace.

Suppose that there are initially *N* agents at the base. Let $n_{i}$ define the *i*th agent (i∈{1,2,…,N}). Let $n_{i}\in R^{3}$ define the 3D location of $n_{i}$, where i∈{1,2,…,N}. Let $n_{i}\left(k\right)\in R^{3}$ indicate $n_{i}$ at sample step *k*. The sampling interval between sample steps *k* and k+1 is dt seconds.

Because the 3D bounded workspace contains unknown obstacles, it is not possible to derive the number of agents required to cover the entire workspace. Thus, we assume that *N* is sufficiently large to cover the entire workspace. Section 5.1.3 handles the exceptional case in which *N* is not sufficiently large to cover the full workspace.

The motion model of each agent $n_{i}$ is

(1) $$\begin{array}{c} n_{i}\left(k+1\right)=n_{i}\left(k\right)+v_{i}\left(k\right)\times dt. \end{array}$$

Here, $v_{i}\left(k\right)$ defines the velocity of $n_{i}$ at sample step *k*. In our paper, each agent moves based on local interaction with its neighbors. Section 5.3 presents how we set $v_{i}\left(k\right)$ in this paper.

The motion model in (1) has been widely applied in the literature on multi-agent controls [29,30,31,32,33,34,35,36,37]. Let MaxS define the maximum speed of $n_{i}$. This implies that $MaxS\geq ∥v_{i}\left(k\right)∥$ for all i,k.

We acknowledge that (1) does not consider the motion constraints on an agent. Our paper is based on generation of multiple agents’ paths, i.e., weconsider high-level control of multi-agent systems.

For instance, consider an agent such as an Autonomous Underwater Vehicle (AUV) with non-holonomic constraints. Along the generated path, each end point of a straight line is set as the waypoint for an agent. With the waypoints set, we can utilize the path following controller in [38,39] while considering the dynamics of the agent to ensure that an agent visits the waypoints. How to ensure that an agent with motion constraints move along the waypoints is beyond the scope of the present paper.

Let the *sense-communicate range* $r^{cs}$ be defined as the smaller value between the maximum sensing range and the maximum communication range. According to this definition, if $∥n_{i}-n_{j}∥\;<r^{cs}$, then $n_{i}$ and $n_{j}$ can sense each other while communicating with each other.

We say that a puck is *detected* by $n_{i}$ in the case where the relative distance between a puck and $n_{i}$ is less than the sense-communicate range $r^{cs}$. Therefore, our aim is to ensure that every puck is within a distance $r^{cs}$ from at least one agent. In this way, every puck can be found by the deployed agents in a provably complete manner.

Let L(n,m) define a straight line segment with the two end points being two agents, say, n and m. We say that L(n,m) is *collision-free* when L(n,m) does not cross an obstacle. This implies that an agent can safely travel along L(n,m) without colliding with any obstacles.

Let I=(V(I),E(I)) define the graph presenting the networked system. Every node in V(I) indicates an agent. An edge, say $\left\{n_{i},n_{j}\right\}\in E\left(I\right)$, indicates that $n_{i}$ and $n_{j}$ are neighbors. Here, we say that two agents $n_{i}$ and $n_{j}$ are *neighbors* in the case where the following conditions are met:

1. $L(n_{i},n_{j})$ is collision-free;
2. $∥n_{i}-n_{j}∥\;<r^{cs}$, i.e, $n_{i}$ and $n_{j}$ can sense each other while communicating with each other.

Let $N(n_{i})$ indicate the neighbors of an agent $n_{i}$.

Let $S(n_{i})$ define the sphere with radius $r^{cs}$ that has its center located at $n_{i}$. Here, $S(n_{i})$ is called the *sensing sphere* of $n_{i}$. We assume that a puck inside $S(n_{i})$ is detected by $n_{i}$. Let $\partial S(n_{i})$ specify the boundary of $S(n_{i})$.

On $\partial S(n_{i})$, we uniformly generate *Q* points. For instance, we discuss the generation of Q=72×72 points on $\partial S(n_{i})$. (This method is utilized in Section 6). Let ϕ denote the yaw angle such that ϕ∈[π/36,2×π/36,3×π/36…,2π]. In addition, let θ denote the elevation angle such that θ∈[π/36,2×π/36,3×π/36…,2π]. We define fp(ψ,θ) as

(2) $$\begin{array}{c} \mathrm{fp}\left(\psi ,\theta \right)=R\left(\psi ,\theta \right){\left[r,0,0\right]}^{T}. \end{array}$$

Here,

(3) $$\begin{array}{c} R(\psi ,\theta )=R(\psi )R(\theta ) \end{array}$$

where

(4) $$\begin{array}{c} R\left(\psi \right)=\left(\begin{array}{ccc} \cos(\psi ) & -\sin(\psi ) & 0\\ \sin(\psi ) & \cos(\psi ) & 0\\ 0 & 0 & 1 \end{array}\right) \end{array}$$

and

(5) $$\begin{array}{c} R\left(\theta \right)=\left(\begin{array}{ccc} \cos(\theta ) & 0 & \sin(\theta )\\ 0 & 1 & 0\\ -\sin(\theta ) & 0 & \cos(\theta ) \end{array}\right). \end{array}$$

In (4), R(ψ) presents the rotation of angle ψ centered at the z-axis. In (5), R(θ) presents the rotation of angle θ centered at the y-axis.

As we add $n_{i}$ to fp(ψ,θ), we obtain the global position of a point on $\partial S(n_{i})$. Note that ψ and θ are respectively selected from among 36 values. Therefore, we derive Q=72×72 points on $\partial S(n_{i})$.

An *open point* of $n_{i}$ denotes a point among *Q* points of $n_{i}$ such that a straight line segment connecting $n_{i}$ and the point does not cross any obstacles. In practice, open points can be detected by an agent’s local sensors with range $r^{cs}$. For instance, lidar sensors on $n_{i}$ can be used to derive the open points of $n_{i}$.

Consider a case where multiple sensing rays with range $r^{cs}$ are emitted from an agent in order to sense its surroundings. As long as a ray does not cross an obstacle, it can travel a distance $r^{cs}$. In this case, the ray is related to an open point.

A *frontier point* $F(n_{i})$ denotes an open point of $n_{i}$ which exists outside $S(n_{j})$ for all j≠i. As Q→∞, the density of frontier points on $\partial S(n_{i})$ increases. Let *frontierInf* define the set of frontier points as Q→∞.

A frontierInf is on the border between ${⋃}_{i\in \{1,2,\ldots ,N\}}S\left(n_{i}\right)$ and an open space covered by no sensing spheres. If every agent has no frontierInf, then all agents’ sensing spheres cover the entire open space.

### Assumptions for Each Agent

Every agent $n_{i}$ (i∈{1,2,…,N}) is assumed to satisfy the following:

(A1) $n_{i}$ measures the relative position of its neighbor agent with its local sensors;
(A2) $n_{i}$ stores the relative position of its frontier point in $F(n_{i})$;
(A3) Every puck inside $S(n_{i})$ is detected by $n_{i}$.

We consider an agent which can measure the relative position of its neighbor agent utilizing proximity sensors. For example, an agent *n* emits signal pings for measuring its neighbor agent. Suppose that the pings emitted from *n* are reflected from its neighbor agent, say, m∈N(n). In this case, *n* can estimate the elevation angle, azimuth angle, and range to *m* through the 3D multiple signal classification (MUSIC) algorithm [40]. Therefore, Assumption (A1) is viable.

We next show that Assumption (A2) is viable. Recall that a frontier point $F(n_{i})$ denotes an open point of $n_{i}$, which exists outside $S(n_{j})$ for all j≠i. Because the radius of a sensing sphere is $r^{cs}$, the sensing sphere of *n* cannot intersect with that of any other agent which is more than two hops away from *n*.

Under Assumption (A1), an agent *n* can access the relative position of m∈N(n). Moreover, m∈N(n) can access the relative position of its neighbor agent, say, $m^{\prime }\in N\left(m\right)$, as *n* and $m^{\prime }$ are two hops distant from each other. The relative vector from *n* to $m^{\prime }$ is obtained by adding the following two vectors:

(1) the vector from *n* to *m*
(2) the vector from *m* to $m^{\prime }$

In this way, *n* can derive the relative position of an agent which is two hops distant from *n*.

Because $n_{i}$ can measure its adjacent obstacles with its local sensors, $n_{i}$ calculates the relative position of its open point. Therefore, $n_{i}$ can determine an open point which exists outside the sensing sphere of any other agent. In other words, $n_{i}$ can derive the relative position of a frontier point in $F(n_{i})$. Accordingly, Assumption (A2) is viable.

## 5. 3D Multi-Agent Foraging Strategy

This section proposes our 3D multi-agent foraging strategy composed of search and collection steps.

### 5.1. Provably Complete Puck Search

This subsection describes solving the following problem. *Generate a network covering the entire 3D cluttered unknown workspace such that no coverage holes remains while maintaining network connectivity.*

It is considered that there are no more coverage holes when every puck in the 3D cluttered workspace has been detected.

#### 5.1.1. Distributed Generation of a Spanning Tree

As the first step in solving the above problem, we introduce a distributed Breadth First Search (BFS) algorithm, inspired by [41]. In [41], a distributed algorithm introduced to ensure that a node in the network can guide a moving object across the network to the goal. Algorithm 2 in [41] can be applied to make a spanning tree *T* rooted at an agent.

The goal sensor in Algorithm 2 of [41] represents the root in Algorithm 1. In Algorithm 1, each agent *n* stores and updates $hops_{g}\left(n\right)$, which indicates the hop distance to the root, say, $n_{r}$. The root $n_{r}$ initializes $hops_{g}\left(n_{r}\right)=0$. Initially, every other agent initializes $hops_{g}\left(n\right)=\infty$, where $n\neq n_{r}$. In Algorithm 1, ancestor(n) denotes the set of ancestors of *n*.
**Algorithm 1** Distributed BFS algorithm to generate a spanning tree *T*1:Every agent *n* contains $hops_{g}\left(n\right)$, which indicates the hop distance to the root;2:Every agent *n* contains ancestor(n)={};3:The root $n_{r}$ sets $hops_{g}\left(n_{r}\right)=0$ initially;4:One initially sets $hops_{g}\left(n\right)=\infty$ where $n\neq n_{r}$;5:Initially, $n_{r}$ transmits $ancestor(n_{r})$ and $hops_{g}\left(n_{r}\right)$ to its neighbors;6:**repeat**7:   *n* ← every agent;8:   **if** the agent *n* receives ancestor(m) and $hops_{g}\left(m\right)$ from its neighbor, say m∈N(n)       **then**9:      The agent *n* updates its hop distance information using (6);10:     **if** $hops_{g}\left(n\right)$ becomes $hops_{g}\left(m\right)+1$ under (6) **then**11:        The agent *n* updates ancestor(n) using (7);12:        The agent *n* broadcasts $hops_{g}\left(n\right)$ and ancestor(n) to its neighbors;13:     **end if**14:   **end if**15:**until** $hops_{g}\left(n\right)\neq \infty$ for all *n*;

Initially, the root $n_{r}$ sends its hop distance information $hops_{g}\left(n_{r}\right)$ and $ancestor(n_{r})$ to its neighbors. Suppose that an agent *n* receives a hop distance message from its neighbor, say, m∈N(n). Then, *n* updates its hop distance information under

(6) $$\begin{array}{c} hops_{g}\left(n\right)=min\left(hops_{g}\left(m\right)+1,hops_{g}\left(n\right)\right). \end{array}$$

When $hops_{g}\left(n\right)$ becomes $hops_{g}\left(m\right)+1$ under (6), the ancestor set (ancestor(n)) of *n* is updated:

(7) $$\begin{array}{c} ancestor(n)=\{ancestor(m),m\}. \end{array}$$

This implies that the set ancestor(n) is generated by adding *m* to the set ancestor(m). When $hops_{g}\left(n\right)$ becomes $hops_{g}\left(m\right)+1$ under (6), *n* broadcasts the updated $hops_{g}\left(n\right)$ and ancestor(n) to its neighbors.

The tree *T* is built until $hops_{g}\left(n\right)\neq \infty$ for all *n*. In [41,42], it was proved that the number of message broadcasts of each agent in this algorithm is one. This implies that Algorithm 1 has a computational complexity of O(1).

#### 5.1.2. Distributed Puck Search Protocol

In order to find every puck in the 3D bounded workspace, we propose a distributed puck search protocol (Algorithm 2). Algorithm 2 expands the agent network coverage while maintaining the network connectivity until no coverage holes remain in the workspace. For no coverage holes to remain, it must be the case that every puck has been sensed by at least one agent.

In Algorithm 2, all agents are initially at the base. Then, every agent moves one after the other such that whenever each agent turns on its local sensors at its designated position (the target point in Algorithm 2) the unsensed open space is reduces gradually.

In Algorithm 2, all agents are initially at the base. Therefore, every agent is a neighbor to $n_{1}$. In the initial phase of Algorithm 2, $n_{1}$ initiates its local sensors with range $r^{cs}$ while staying at the base. Accordingly, frontier points of $n_{1}$ are formed using the approach in Section 4.
**Algorithm 2** Distributed puck search protocol for all agents*N* agents are located at the base, thus every agent is a neighbor to $n_{1}$;AgentsWithPucks={};The agent $n_{1}$ is at the base’s entrance;The agent $n_{1}$ initiates its local sensors with range $r^{cs}$, and frontier points of $n_{1}$ are formed;i=2;**repeat**   By running Algorithm 1 in Section 5.1.1, begin generation of *T* rooted at $n_{i}$;   Algorithm 1 runs until an agent, say $n^{\prime }$, receives a hop distance message from its neighbor in $N(n^{\prime })$ and a frontier point exists at $n^{\prime }$;   **if** $n_{i}$ cannot find an agent with a frontier point **then**     This puck search algorithm is finished;   **end if**   $P=path(n_{1},n^{\prime })$ in Algorithm 3;   Since $n_{i}$ is a neighbor to $n_{1}$ initially, the path information *P* is transmitted to $n_{i}$;   The agent $n_{i}$ moves along the path to $n^{\prime }$;   Once $n_{i}$ reaches $n^{\prime }$, one frontier point on $n^{\prime }$, say ${tgt}_{n_{i}}$, is set as target point of $n_{i}$;   The agent $n_{i}$ moves to ${tgt}_{n_{i}}$;   **if** the agent $n_{i}$ reaches ${tgt}_{n_{i}}$ **then**     The agent $n_{i}$ initiates its local sensors with range $r^{cs}$, and detects its neighbors;     Frontier points of $n_{i}$ are formed using the approach in Section 4;     **if** $n_{i}$ detects a puck **then**        $AgentsWithPucks.append(n_{i})$;     **end if**   **end if**   i=i+1;**until** i==N;

Algorithm 2 applies to all agents. In Algorithm 2, every agent initiates its maneuver in the following order: $n_{2}\to n_{3}\cdots \to n_{N}$. Whenever a new agent, say $n_{i}$, initiates movement, it continues moving until meeting its target point. After meeting the target point, $n_{i}$ initiates its local sensors with range $r^{cs}$. This sensor initialization constructs frontier points for $n_{i}$, as addressed in Section 4. Whenever each agent reaches its target point, the unsensed open space is gradually reduced. This iterates until every agent is located at its designated target point.

In Algorithm 2, AgentsWithPucks denotes the set of agents that have detected a puck. Whenever an agent initiates its local sensor, it may detect a puck. When an agent detects a puck, that agent is added to the agent set AgentsWithPucks. For instance, if an agent $n_{i}$ detects a puck, $AgentsWithPucks.append(n_{i})$ is performed as presented in Algorithm 2.

In Algorithm 2, *T* rooted at $n_{i}$ is generated by running Algorithm 1 from Section 5.1.1. Algorithm 1 runs until an agent, say $n^{\prime }$, receives a hop distance message from its neighbor, say $m\in N(n^{\prime })$, and $n^{\prime }$ has a frontier point. In this case, $n^{\prime }$ needs to let $n_{i}$ access a path to $n^{\prime }$. Algorithm 3 is used to make $n_{i}$ access a path to $n^{\prime }$. In this algorithm, the initial path $P=\{n^{\prime }\}$ is generated from $n^{\prime }$. Algorithm 3 iteratively appends a parent node to *P* until $n_{1}$ receives *P*. Because $n_{i}$ is initially a neighbor to $n_{1}$, the path information *P* is transmitted to $n_{i}$.
**Algorithm 3** $P=path(n_{1},n^{\prime })$The agent $n^{\prime }$ broadcasts $P=\{n^{\prime }\}$ to its parent $p(n^{\prime })$ in *T*;**repeat**   **if** an agent *n* receives the path information *P* **then**     P.append(n);     *P* is transmitted to p(n);   **end if****until** $n_{1}$ receives *P*;Return *P*;

In Algorithm 2, $n_{i}$ travels along the path in *T* to reach $n^{\prime }$. For convenience, let *P* denote this path. Next, we present how $n_{i}$ travels along *P*. According to the definition of an edge E(I), *P* is collision-free for $n_{i}$. Furthermore, the length of every line segment of *P* is shorter than $r^{cs}$. As $n_{i}$ encounters an agent in *P*, $n_{i}$ can move towards the next agent in *P* utilizing its local sensors. As $n_{i}$ encounters an agent in *P*, say $n_{l}$, $n_{i}$ accesses the next agent in *P* utilizing its local sensors. This is viable due to Assumption (A1). Note that global positioning is not required for this local maneuver.

As $n_{i}$ encounters the last agent in *P*, $n_{i}$ can move to its target point ${tgt}_{n_{i}}$. This maneuver is performed using the local sensors of $n_{i}$, based on Assumption (A2). After $n_{i}$ encounters its target point ${tgt}_{n_{i}}$, $n_{i}$ initiates its local sensors. When $n_{i}$ initiates its local sensors, several frontier points inside $S(n_{i})$ are removed.

Next, we verify that this removal is viable. Consider a case where a frontier point in $F(n_{j})$ is inside $S(n_{i})$. Because the relative distance between $n_{j}$ and a frontier point in $F(n_{j})$ is $r^{cs}$, $n_{j}$ is a neighbor to $n_{i}$. Under Assumption (A1), $n_{i}$ measures the relative position of $n_{j}$. The relative vector from $n_{i}$ to a frontier point in $F(n_{j})$ is obtained by adding the following two vectors:

(1) The vector from $n_{i}$ to $n_{j}$
(2) The vector from $n_{j}$ to the frontier point in $F(n_{j})$

The above two vectors are accessible based on Assumptions (A1) and (A2). As $n_{i}$ initiates its local sensors, several frontier points inside $S(n_{i})$ are removed. Thereafter, $n_{i+1}$ is deployed, finds a frontier point, and travels along *T* until meeting the frontier point. This continues until Algorithm 2 ends.

Algorithm 2 is complete if i==N or if no frontier point is found using *T*. In Algorithm 2, an agent $n_{i}$ travels along a path in *T* until meeting ${tgt}_{n_{i}}$. Consequently, we have the following Theorem 1.

**Theorem** **1.** *Every agent maneuvers utilizing Algorithm 2. When an agent $n_{i}$ travels along a path in T until meeting ${tgt}_{n_{i}}$, collision avoidance is assured. As $n_{i}$ travels along a path in T, say P, $n_{i}$ moves based on local interaction with sense-communication range $r^{cs}$.*

**Proof.**  Agent $n_{i}$ travels along a path in *T*, say *P*, until meeting ${tgt}_{n_{i}}$. Let $P=\{m_{1}\to m_{2}\to \cdots \to m_{end}\}$ define the agents along the path. After $n_{i}$ encounters $m_{j}$ (j≤end−1), $n_{i}$ moves to $m_{j+1}$. Furthermore, after $n_{i}$ encounters $m_{end}$, $n_{i}$ moves to ${tgt}_{n_{i}}$. The maneuver of $n_{i}$ is performed using the local sensing measurements of $n_{i}$ under Assumptions (A1) and (A2).

According to the definition of an edge E(I), *P* is collision-free for $n_{i}$. Furthermore, the length of every line segment of *P* is shorter than its sense-communication range $r^{cs}$. Thus, the theorem is proved.    □

Consider an ideal case where Q=∞. We then have Theorem 2. Theorem 2 proves that when the 3D network is fully constructed utilizing Algorithm 2, then no coverage holes remain. When no coverage holes exist, every puck in the 3D cluttered workspace is detected.

**Theorem** **2.** *If a frontierInf cannot be detected using the 3D network, then the open space is entirely covered by all sensing spheres such that no coverage holes exist.*

**Proof.**  Utilizing the transposition rule, we can prove the following statement. If an open space which exists outside of every sensing sphere exists, then a frontierInf associated with the space can be detected.

We consider a bounded and closed 3D workspace. Consider the case where an open space which is outside of every sensing sphere exists. Let *O* specify this uncovered open space. A frontierInf is on the border between ${⋃}_{i\in \{1,2,\ldots ,N\}}S\left(n_{i}\right)$ and an open space covered by no sensing spheres. Therefore, at least one agent, say $n_{j}$, has a frontierInf intersecting the boundary of *O*. Utilizing Theorem 1, $n_{j}$ is connected to $n_{r}$. Accordingly, we can find this frontierInf utilizing *T*.    □

Theorem 2 proves that utilizing Algorithm 2, every puck in the 3D cluttered workspace is detected when we cannot detect a frontierInf using the 3D network. However, even in the case where a frontierInf is detected, we require the base to have at least one remaining agent to cover the frontierInf. Thus, Algorithm 2 can only lead to detection of all pucks if *N* is sufficiently large to cover the entire workspace.

#### 5.1.3. Exception Handling

Up to now, we have considered the case in which all agents begin moving from the base. In practice, it may be the a case that *i* reaches *N* when Algorithm 2 is complete. This implies that there may not be sufficient agents in the base. In this case, it may be necessary to deploy new agents from a new point, which can be far from the base.

These new agents run Algorithm 2 starting from their initial locations. As time passes, the network built by new agents, say *I*, meets with the network built by other agents, say, $I^{c}$. This implies that an agent in *I* becomes a neighbor to an agent in $I^{c}$. In this case, the network built by *I* is merged with that built by $I^{c}$. Then, Algorithm 1 in Section 5.1.1 is run to cover the merged network. Using Theorem 2, this merged network keeps expanding until no coverage holes remain. Note that our distributed search strategy is provably complete in detecting all pucks in the 3D cluttered bounded workspace.

### 5.2. Collection of All Pucks

Next, we address the collection step, which involves collecting all of the pucks and returning them to the base. Every puck needs to be collected and returned to $n_{1}$, which is located at the base. In the collection step, no global localization or global communication can be used. Therefore, the agents need to collect the pucks while maintaining network connectivity.

To maintain network connectivity during the collection process, agents are divided into *carrier agents* and *guiding agents*. A carrier agent is assigned to a puck and moves along the path to its designated puck in order to carry the puck. Every agent on a path to a puck is called a guiding agent, because the guiding agents’ task is to guide the carrier agents which bring the puck to the base.

Guiding agents are utilized for agent maneuvers, as our foraging strategy does not depend on global localization. A carrier agent can reach a puck by iteratively visiting guiding agents along the path to the puck. Note that local interaction between agents is relied on to ensure that the carrier agents move along the path. In this way, a carrier agent can carry its assigned puck to the base by iteratively visiting guiding agents. In summary, every agent on a path to a puck is used as a guiding agent to guide the carrier agents in reaching the pucks and bringing them to the base.

#### 5.2.1. All Agents Except for the Guiding Agents Rendezvous at the Base

When the 3D workspace has been entirely sensed utilizing Algorithm 2, every puck is sensed by at least one agent. Under Algorithm 2, every puck in the 3D workspace is detected by an agent in AgentsWithPucks.

A path can now be generated from the base to every agent in AgentsWithPucks. Every agent on the path to an agent in AgentsWithPucks is utilized as a waypoint for guiding agents in moving between the base and the pucks.

Let $path(n_{1},n)\in T$ define a path from $n_{1}$ to *n*. Every agent on $path(n_{1},n_{p})$ for all $n_{p}\in AgentsWithPucks$ is called a *guiding agent*. Every agent except for the guiding agents is called a *carrier agent*, and has the task of carrying a puck to the base.

We first make all agents except for guiding agents rendezvous at the base, as they now need to work as carrier agents. Algorithm 4 is used to make all agents except for guiding agents rendezvous at the base.

Algorithm 4 was inspired by the distributed rendezvous control presented in [43]. The distinction between our article and [43] is that here we need to make all agents except for guiding agents rendezvous at the base. The guiding agents need to stay at their position in order to guide the carrier agents in moving between the base and the pucks. Algorithm 4 presents the gathering algorithm used to make all agents except for the guiding agents rendezvous at the base.
**Algorithm 4** Distributed gathering strategy for all agents except for guiding agents1:Let $n_{1}$ denote an agent located at the base;2:Apply Algorithm 1 in Section 5.1.1 for generating a tree *T* rooted at $n_{1}$;3:**repeat**4:   *n* ← every agent, other than guiding agents;5:   **if** *n* is a leaf in *T* **then**6:     The agent *n* moves along a path to $n_{1}$ using ancestor(n) (*n* keeps visiting its ancestors using *T*);7:   **else if** *n* is not a leaf in *T* **them**8:     **if** *n* encounters all its descendants except for guiding agents **then**9:        The agent *n* moves along a path to $n_{1}$ using ancestor(n) (*n* keeps visiting its ancestors using *T*);10:     **end if**11:   **end if**12:**until** every agent, other than guiding agents, visits $n_{1}$;

Assume that an agent, say *n*, is not a guiding agent. Suppose that *n* has a guiding agent, say $n_{g}$, as its ancestor in *T*. In this case, *n* can visit agents along the path to the base, which contains $n_{g}$. This implies that *n* utilizes $n_{g}$ as a “waypoint” along the path to the base.

Under Algorithm 4, an agent waits until it has met all its descendants except for guiding agents. Because $n_{g}$ is a guiding agent, $n_{g}$ must not move at all under Algorithm 4. Thus, $p(n_{g})$ removes $n_{g}$ from its descendants list; $p(n_{g})$ starts moving after all of its descendants other than $n_{g}$ have met $p(n_{g})$. In this way, all agents except for guiding agents rendezvous at the base. When these agents have gathered at the base, they are used as carrier agents for carrying the pucks to the base.

To implement Algorithm 4, each agent stores its parent in *T*, its descendant list, and its guiding agents. The following theorem shows that Algorithm 4 is distributed and that every agent is connected to $n_{1}$ while it moves. Moreover, collision avoidance is assured while every agent is moving.

**Theorem** **3.** *Algorithm 4 is distributed, and every agent is connected to the root $n_{1}$ while it moves. Moreover, collision avoidance is assured while every agent is moving.*

**Proof.**  Suppose that an agent *n* has been visiting agents along the path to the root $n_{1}$ in Algorithm 4. For convenience, let PATH indicate this path. Suppose that PATH consists of a set of agents $p_{1}\to p_{2}\to p_{3}\cdots \to p_{end}$ in this order. Here, $p_{end}$ is $n_{1}$.

According to the definition of an edge E(I), PATH is collision-free for *n*. Furthermore, the length of every line segment of PATH is shorter than sense-communication range $r^{cs}$.

We can show that $p_{i}$, where i∈{1,2,…,end−1}, starts moving only after *n* meets $p_{i}$. Any agent other than a leaf starts moving only after all of its descendants except for guiding agents in *T* have met it. Any agent in PATH is an ancestor of *n*. Therefore, $p_{i}$ starts moving only after *n* has met $p_{i}$.

If *n* has just met $p_{i}$, then *n* can sense $p_{i+1}$ utilizing local sensors. Because *n* moves based on local sensing measurements, Algorithm 4 is distributed.

Next, we show that every agent remains connected to the root during its maneuver. Suppose that *n* has just encountered $p_{i}$ and starts moving towards $p_{i+1}$. Then, *n* is connected to $p_{i+1}$. All agents in $p_{i+1}\to p_{i+2}\to p_{i+3}\cdots \to p_{end}=n_{1}$ remain stationary. Therefore, $p_{i+1}$ is connected to $n_{1}$. Because *n* is connected to $p_{i+1}$, *n* is connected to $n_{1}$. Thus, it is proved that every agent is connected to $n_{1}$ during its movement.    □

#### 5.2.2. Puck Collection Using Carrier Agents

Let $n_{p}$ define an agent in AgentsWithPucks. Then, $path(n_{1},n_{p})$ defines a path from $n_{1}$ to $n_{p}$. Each carrier agent at the base is assigned to a puck and moves along the path to its designated puck in order to carry the puck. For instance, if there are *C* carrier agents in total then we can assign $\frac{C}{∥AgentsWithPucks∥}$ carrier agents to each puck, ensuring that the puck are assigned an identical number of carrier agents.

Every carrier agent utilizes guiding agents as “static waypoints” along the path between the puck and the base. Guiding agents are utilized because our foraging strategy does not depend on global localization. Note that local interaction between agents is used to make the carrier agents move along the path. The local control in Section 5.3 can be applied to make a carrier agent move along the path to its designated puck. When a carrier agent reaches its assigned puck, the carrier agent carries its assigned puck to the base by iteratively visiting its guiding agents. In this way, each carrier agent transports a puck to the base.

Multiple carrier agents can bring the pucks back to the base simultaneously. While carrier agents are moving, they need to avoid colliding with each other. For local collision avoidance, we can apply reactive collision avoidance controls in [44,45,46,47]. Because our paper handles high-level controls of multi-robot systems, developing local collision avoidance controls is not within the scope of this paper.

When all pucks are carried to the base, we need to make all guiding agents rendezvous at the base. Similarly to Algorithm 4, Algorithm 5 can be used to ensure that all guiding agents rendezvous at the base while maintaining network connectivity.
**Algorithm 5** Distributed gathering strategy for guiding agentsLet $n_{1}$ denote an agent located at the base;Apply Algorithm 1 in Section 5.1.1, to generate a tree *T* rooted at $n_{1}$;**repeat**   *n* ← every guiding agent;   **if** *n* is a leaf in *T* **then**     The agent *n* finds a path to $n_{1}$ using ancestor(n);     The agent *n* starts visiting every agent along the path (*n* keeps visiting its ancestors using *T*);    **else if** *n* is not a leaf in *T* **then**     **if** *n* encounters all its descendants **then**        The agent *n* finds a path to $n_{1}$ using ancestor(n);         The agent *n* starts visiting every agent along the path (*n* keeps visiting its ancestors using *T*);     **end if**   **end if****until** every guiding agent visits $n_{1}$;

### 5.3. Control of an Agent Based on Local Interactions

In Algorithms 2, 4 and 5, an agent, say $n_{i}$, travels along a path until meeting the last agent on the path. Let $P=\{m_{1}\to m_{2}\to \cdots \to m_{end}\}$ define the agents on the path. After $n_{i}$ encounters $m_{j}$ (j≤end−1), $n_{i}$ heads towards $m_{j+1}$. We address 3D local controls for $n_{i}$ such that $n_{i}$ travels along *P* until reaching $m_{end}$.

Using the definition of edge in *T*, this path *P* is collision-free and a sense-communication link is established between $m_{j}$ and $m_{j+1}$ in *P*. Along *P*, each end point of a straight line is set as the way point for $n_{i}$.

Local controls for $n_{i}$ are developed in discrete-time systems. Let $n_{i}\left(k\right)$ define the global coordinates $n_{i}$ at sample step *k*. Let *W* define the next waypoint that $n_{i}$ will encounter as $n_{i}$ travels along *P*. Let W define the global coordinates of *W*.

For convenience, let $g=W-n_{i}\left(k\right)$. According to Assumption (A1), $n_{i}$ can measure g utilizing its local sensors. For example, $n_{i}$ emits signal pings for measuring its neighbor agent. Suppose that the pings emitted from $n_{i}$ are reflected from its neighbor agent, say, $m\in N(n_{i})$. In this case, $n_{i}$ can estimate the elevation angle, azimuth angle, and range to *m* through the 3D MUSIC algorithm [40].

Recall that dt denotes the sampling interval of our controls. If ∥g∥ >MaxS×dt, then $v_{i}\left(k\right)$ in (1) is set as

(8) $$\begin{array}{c} v_{i}\left(k\right)=MaxS\times \frac{g}{∥g∥}. \end{array}$$

This implies that $n_{i}$ moves towards W with its maximum speed. If ∥g∥ ≤MaxS×dt, then $n_{i}$ heads towards the next waypoint after W.

Suppose that we consider an agent, such as an AUV, with non-holonomic constraints. Along the AUV’s path *P*, each end point of a straight line is set as a waypoint for the AUV. With the waypoints set, we can utilize the path following controllers in [38,39] while considering the dynamics of the AUV to make the AUV visit the waypoints. How to ensure that an agent with motion constraints moves along waypoints is beyond the scope of the present paper.

## 6. MATLAB Simulation Results

### 6.1. Verification of Algorithm 2 (Puck Search Process)

We first verified the performance of Algorithm 2 (puck search process) with MATLAB simulations. The sampling interval was set as dt=1 second. We utilized Q=72×72 in (3). In addition, we used a cluttered unknown 3D workspace with spherical obstacles. We considered a bounded 3D workspace with a size of 300×300×300 in meters.

The sense-communication range was $r^{cs}=80$ m and the maximum speed MaxS was 5 m/s. The base was located at (30,30,0) in meters.

**Figure 2:**
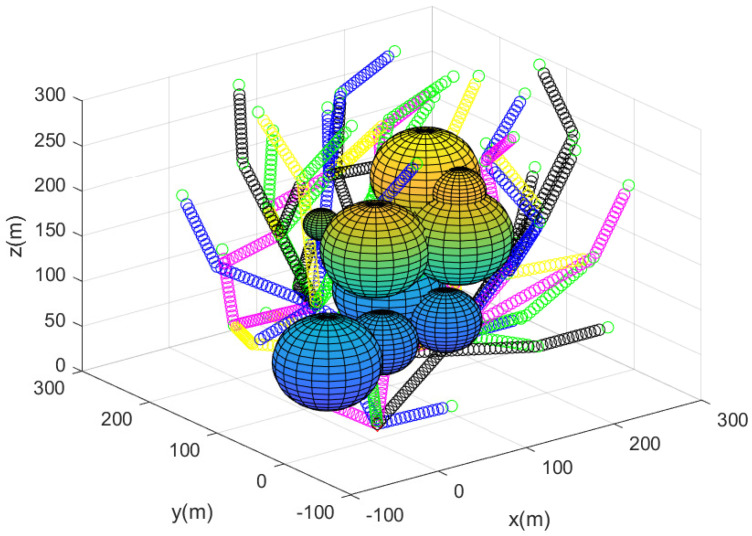
This figure describes the sensor network constructed utilizing Algorithm 2. The path of each agent starting from the base is described by line segments with distinct colors.

Figure 2 describes the agents’ positions after utilizing Algorithm 2 to deploy agents for detecting every puck in the 3D workspace. The spheres represent 3D obstacles in the cluttered bounded workspace. In all, 75 agents begin moving from the base; the 3D position of the agents is described with a small asterisk. In Figure 2, the path of each agent starting from the base is described by line segments with distinct colors.

**Figure 3:**
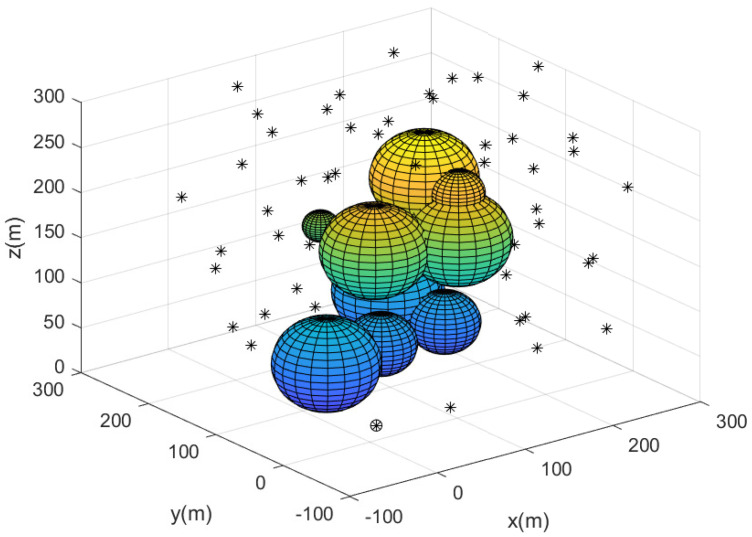
This figure describes the complete sensor network constructed utilizing Algorithm 2. Deployed agents are marked with black asterisks. The base is marked with a circle.

Figure 3 describes the complete sensor network constructed utilizing Algorithm 2. In Figure 3, the deployed agents are marked with black asterisks. The base is marked with a circle. All agents construct the complete 3D network without coverage holes. This implies that every puck in the 3D workspace has been detected by at least one agent.

**Figure 4:**
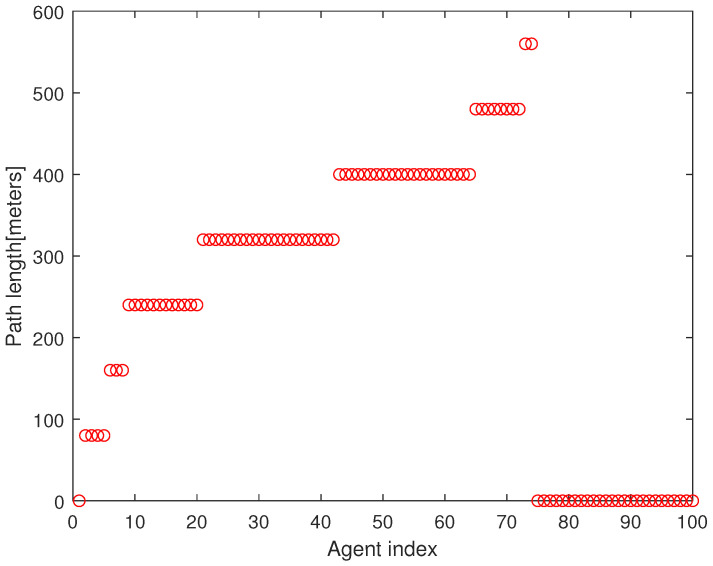
This figure describes the path length of every agent under Algorithm 2. The x-axis presents the agent index and the y-axis presents the path length of the agent until it meets the associated target point.

As a quantitative analysis, Figure 4 presents the path length of every agent under Algorithm 2. The x-axis presents the agent index and the y-axis presents the path length of the agent until it meets the associated target point. In all, 75 agents begin moving from the base. In Algorithm 2, every agent initiates its maneuver in the following order: $n_{2}\to n_{3}\cdots \to n_{N}$. It takes 173 s to run Algorithm 2 using the MATLAB simulations; hence, Algorithm 2 is suitable for real-time applications.

### 6.2. Verification of Algorithm 4 (Distributed Gathering Process)

When the 3D workspace is entirely sensed utilizing Algorithm 2, every puck has been sensed by at least one agent. Suppose that all agents in AgentsWithPucks have detected all pucks in the 3D workspace and that AgentsWithPucks is composed of the following agents ($n_{5},n_{10},n_{15},n_{19},n_{20}$).

**Figure 5:**
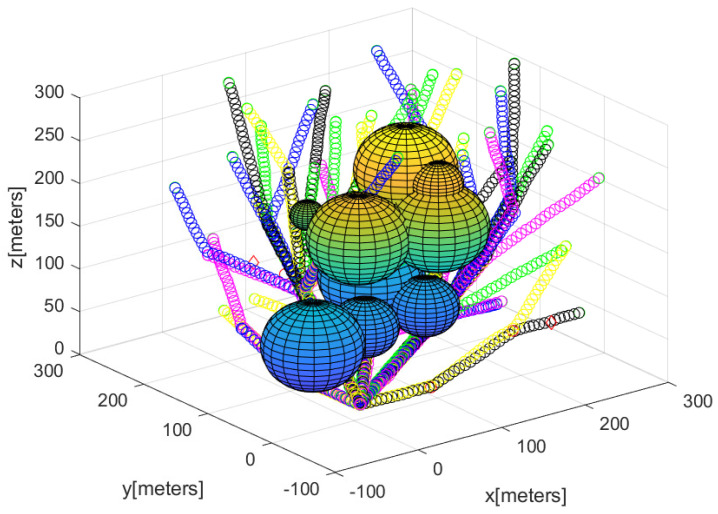
Considering the scenario in Figure 3, the MATLAB simulation result of Algorithm 4 is plotted here. The paths taken by agents, except for guiding agents, to gather at the base are marked by circles with distinct colors.

Algorithm 4 describes how to make all agents except for guiding agents rendezvous at the base in a distributed manner. The MATLAB simulation result of Algorithm 4 is plotted in Figure 5. In Figure 5, the paths taken by the agent other than guiding agents to gather at the base are marked by circles with distinct colors. It takes only 7 s to run Algorithm 4 using the MATLAB simulation. Therefore, Algorithm 4 is suitable for real-time applications.

### 6.3. Verification of the Puck Collection Process

Section 5.2 has addressed the process of collecting all of the pucks in the 3D workspace. We used MATLAB simulations to verify the puck collection process. Suppose that AgentsWithPucks is composed of agents ($n_{5},n_{10},n_{15},n_{19},n_{20}$).

**Figure 6:**
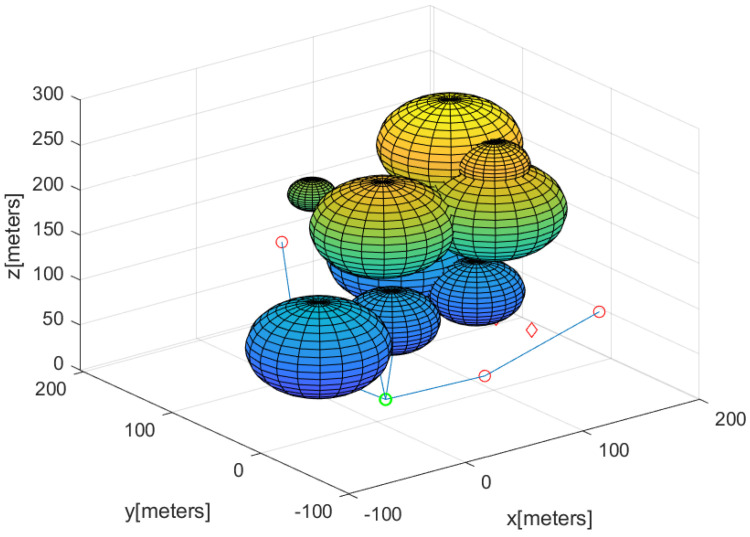
Considering the scenario in Figure 3, this figure describes the positions of the guiding agents after the complete 3D network is built. The base is marked with a green circle. A puck is marked with a red diamond. The path from the base to an agent in AgentsWithPucks is depicted by blue line segments. The guiding agents are plotted by small circles along the blue line segments.

Considering this scenario, Figure 3 and Figure 6 describe the guiding agents after the complete 3D network is built. In this figure, a puck is marked with a red diamond. The base is marked with a green circle. The path from the base to the agent in AgentsWithPucks is described by blue line segments. The guiding agents are described by small circles along the blue line segments. Using these paths, the carrier agent can carry the puck to the base without relying on GPS.

Each carrier agent is assigned to a puck and moves along the path to its designated puck in order to carry the puck. The local control in Section 5.3 can be utilized to make a carrier agent move along the path to its designated puck.

When all pucks have been carried to the base, all guiding agents then need to rendezvous at the base. Algorithm 5 ensures that all guiding agents rendezvous at the base while maintaining network connectivity.

### 6.4. Effect of Changing $R^{Cs}$

**Figure 7:**
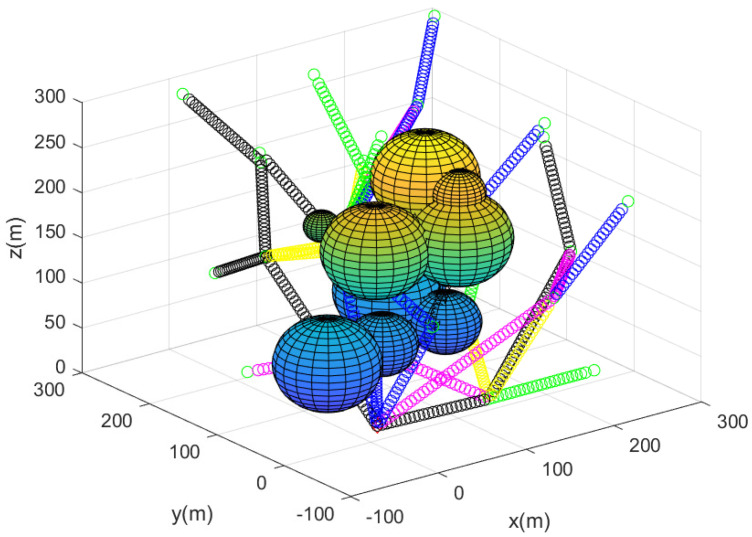
Considering the case where $r^{cs}=120$ m, this figure describes the sensor network constructed utilizing Algorithm 2. The path of each agent starting from the base is described by line segments with distinct colors.

**Figure 8:**
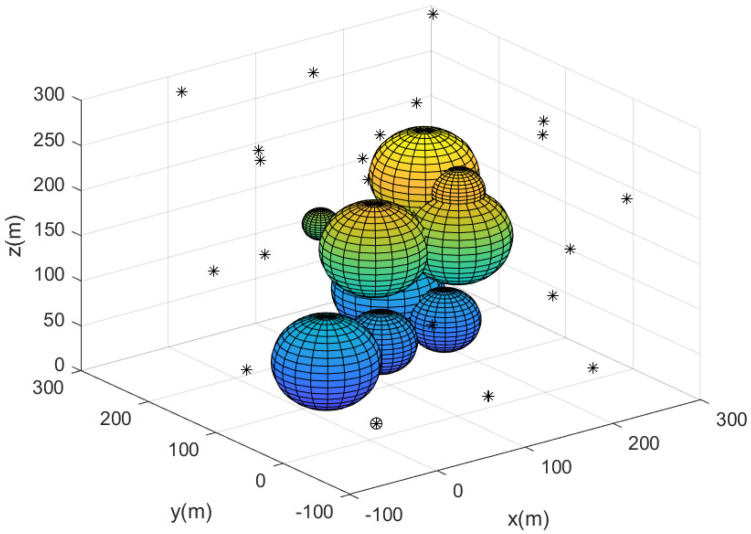
Considering the case where $r^{cs}=120$ m, this figure describes the complete sensor network constructed utilizing Algorithm 2. Deployed agents are marked by black asterisks. The base is marked by a circle.

Up to now, we have used a sense-communication range of $r^{cs}=80$ m. For comparison, we next simulated the case where $r^{cs}=120$ m. Figure 7 describes the sensor network constructed utilizing Algorithm 2. The path of each agent starting from the base is described by line segments with distinct colors. The agents are more sparsely deployed compared to Figure 2, as now $r^{cs}=120$ m. Figure 8 describes the complete sensor network constructed utilizing Algorithm 2; 29 agents begin moving from the base, and the 3D positions of the agents are described by black asterisks.

**Figure 9:**
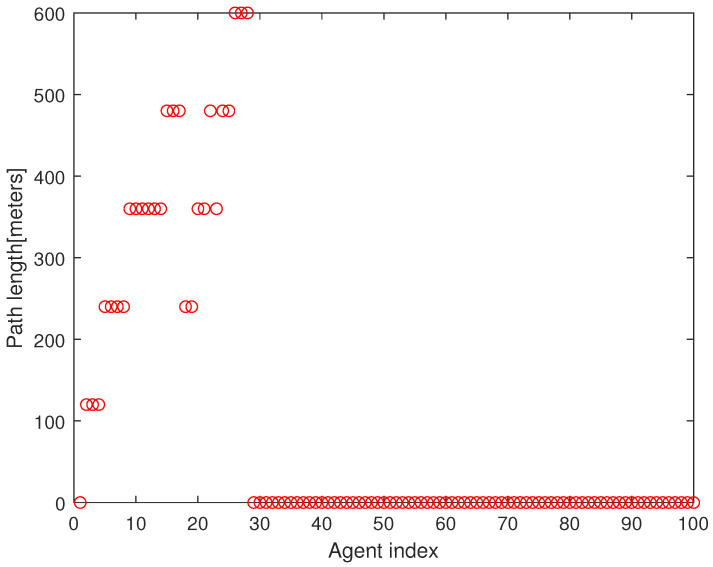
Considering the case where $r^{cs}=120$ m, this figure describes the path length of every agent under Algorithm 2. The x-axis presents the agent index and the y-axis presents the path length of the agent until it meets the associated target point.

As a quantitative analysis, Figure 9 presents the path length of every agent under Algorithm 2. In all, 29 agents begin moving from the base. It takes only 21 s to run Algorithm 2 using MATLAB simulations. Thus, Algorithm 2 is suitable for real-time applications.

**Figure 10:**
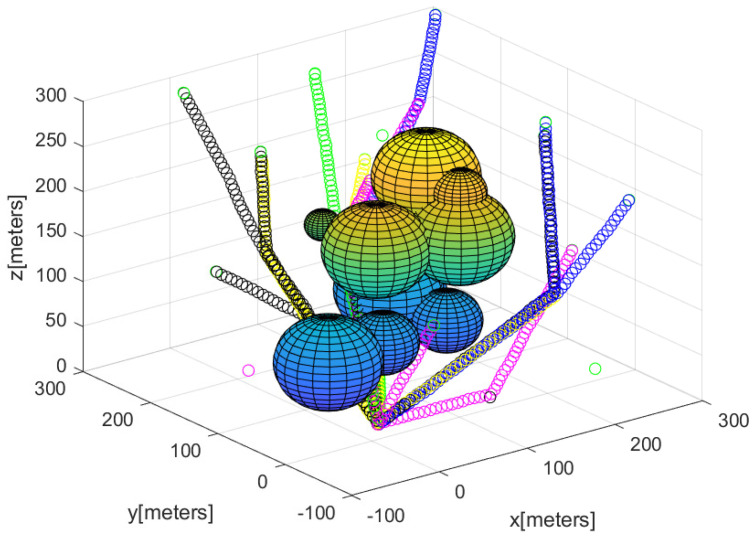
Considering the scenario in Figure 8, this figure plots the MATLAB simulation results of Algorithm 4 for the case where $r^{cs}=120$ m. The path taken by the agents other than guiding agents to gather at the base are marked by circles with distinct colors.

**Figure 11:**
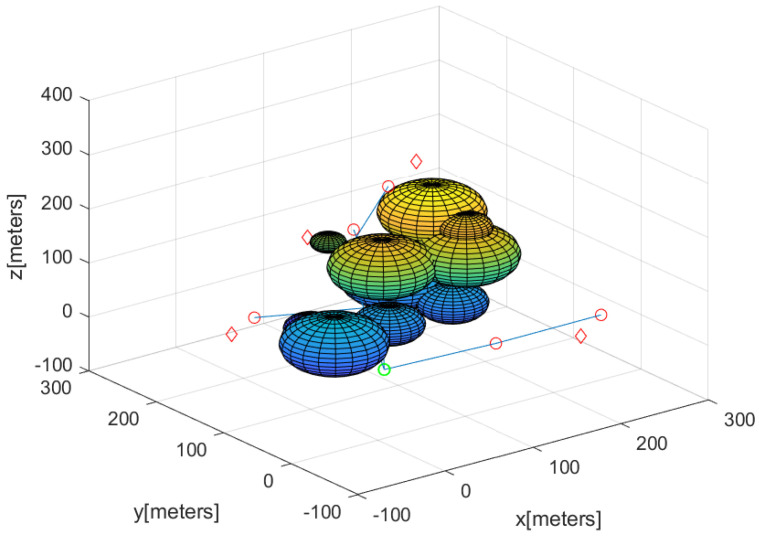
Considering the scenario in Figure 8, this figure describes the positions of the guiding agents after the complete 3D network is built for the case where $r^{cs}=120$ m. A puck is marked by a red diamond. The base is marked by a green circle. The path from the base to an agent in AgentsWithPucks is depicted by blue line segments. The guiding agents are plotted by small circles along the blue line segments.

Considering the scenario in Figure 8, the MATLAB simulation results of Algorithm 4 are plotted in Figure 10. In Figure 10, the paths taken by the agents other than guiding agents to gather at the base are marked by circles with distinct colors. Considering the scenario in Figure 8 and Figure 11, this figure describes the positions of the guiding agents after the complete 3D network is built. In Figure 11, a puck is marked with a red diamond. The path from the base to an agent in AgentsWithPucks is depicted with blue line segments. The guiding agents are plotted by small circles along the blue line segments. Using these paths, carrier agents can carry pucks to the base without relying on GPS.

### 6.5. Effect of Changing Obstacle Environments

**Figure 12:**
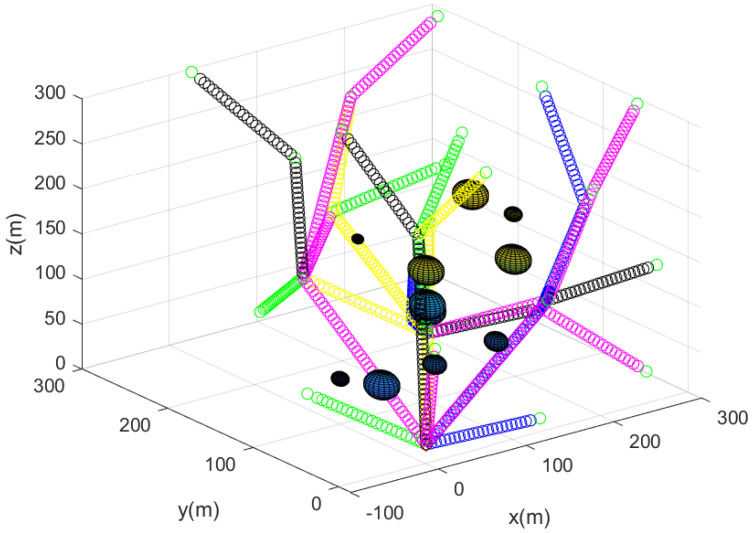
This figure describes the sensor network constructed utilizing Algorithm 2 for a sparse obstacle environment in the case where $r^{cs}=120$ m. The paths of each agent starting from the base are described by line segments with distinct colors.

**Figure 13:**
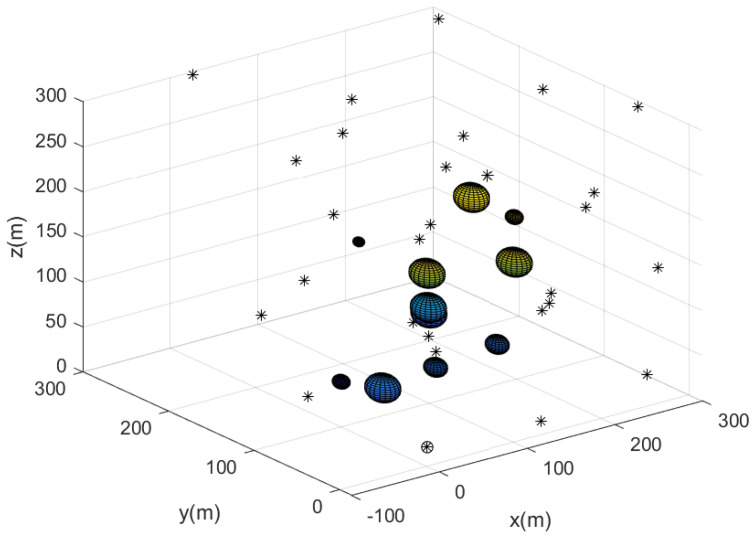
This figure describes the complete sensor network constructed utilizing Algorithm 2 for a sparse obstacle environment in the case where $r^{cs}=120$ m. Deployed agents are marked by black asterisks. The base is marked by a circle.

For comparison, we next used sparse obstacle environments for the case where $r^{cs}=120$ m. Figure 12 describes the sensor network constructed utilizing Algorithm 2. The path of each agent starting from the base is described by line segments with distinct colors. Figure 13 describes the complete sensor network constructed utilizing Algorithm 2. In all, 30 agents begin moving from the base; the 3D position of the agents are described by a black asterisks.

**Figure 14:**
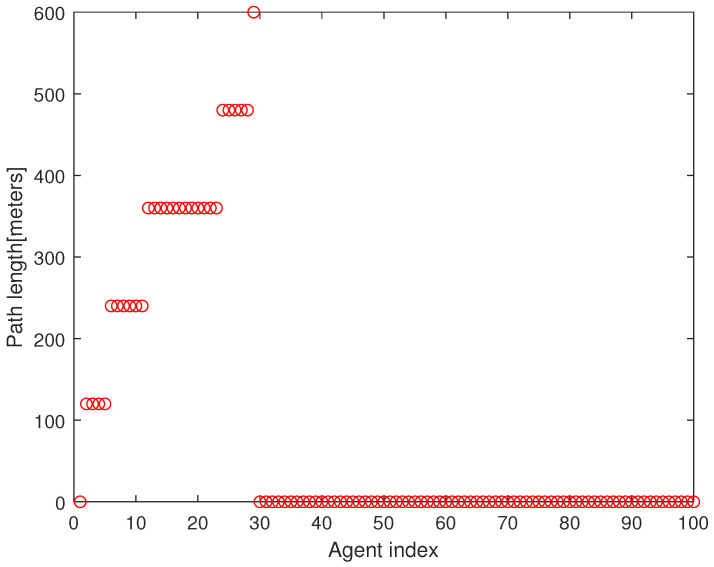
Considering the sparse obstacle environments while setting $r^{cs}=120$ m, this figure describes the path length of every agent under Algorithm 2. The x-axis presents the agent index and the y-axis presents the path length of the agent until it meets the associated target point.

As a quantitative analysis, Figure 14 presents the path length of every agent under Algorithm 2. In all, 30 agents begin moving from the base. It takes only 27 s to run Algorithm 2 using MATLAB simulations. Therefore, Algorithm 2 is suitable for real-time applications.

**Figure 15:**
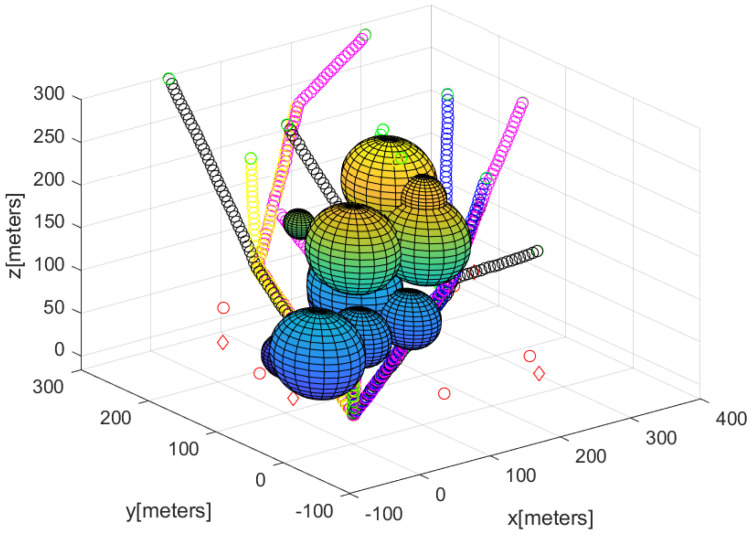
Considering the scenario in Figure 13, this figure plots the MATLAB simulation results of Algorithm 4. The paths taken by agents other than guiding agents to gather at the base are marked by circles with distinct colors.

**Figure 16:**
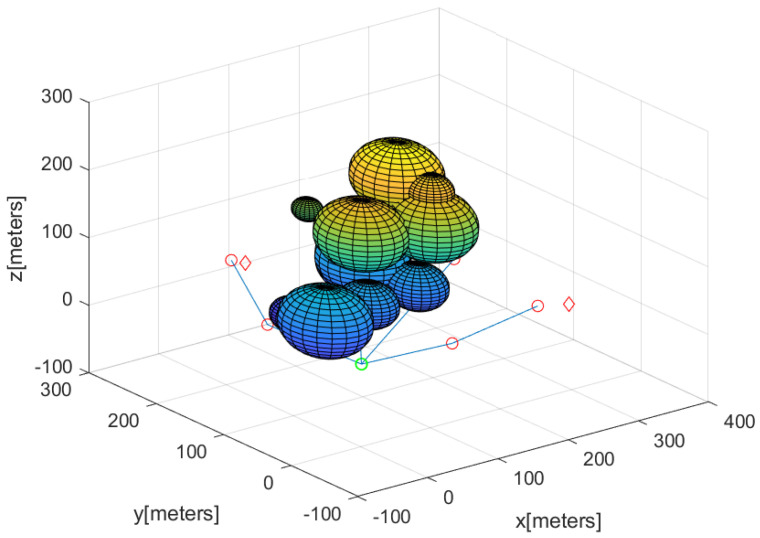
Considering the scenario in Figure 13, this figure describes the positions of guiding agents after the complete 3D network is built. A puck is marked by a red diamond. The base is marked by a green circle. The path from the base to an agent in AgentsWithPucks is depicted by blue line segments. The guiding agents are plotted by small circles along the blue line segments.

Considering the scenario in Figure 13, the MATLAB simulation results of Algorithm 4 are plotted in Figure 15. Considering the scenario in Figure 13, Figure 16 describes the positions of the guiding agents after the complete 3D network is built. In Figure 16, a puck is marked by a red diamond. The path from the base to an agent in AgentsWithPucks is depicted by blue line segments. The guiding agents are plotted by small circles along the blue line segments. Using these paths, carrier agents can carry pucks to the base without relying on GPS.

## 7. Conclusions

In this article, we have proposed a 3D multi-agent foraging strategy composed of puck search and collection. The proposed 3D multi-agent foraging strategy is summarized as follows. In the search process, agents move one by one to expand the network until the 3D cluttered workspace is fully covered. When the bounded workspace is entirely covered by deployed agents, every puck is detected by an agent’s local sensors. Next, we generate a path from the base to every puck. All agents along a path to a puck are set as guiding agents. Algorithm 4 then runs to let all agents except for guiding agents rendezvous at the base. Thereafter, every carrier agent at the base is assigned to a puck and travels along the path to its designated puck in order to collect the puck. When a carrier agent reaches its assigned puck, the carrier agent can carry its assigned puck to the base by iteratively visiting guiding agents. When all pucks have been carried to the base, Algorithm 5 runs to make all agents rendezvous at the base while maintaining network connectivity.

In our paper, agents are deployed such that the sensing spheres (spheres with radius $r^{cs}$ centered at an agent) of all agents cover the entire workspace. In this article, we prove that every puck in the 3D cluttered bounded workspace is detected in finite time. To the best of our knowledge, this article is novel in letting multiple agents perform foraging and puck gathering while satisfying network connectivity requirements in a cluttered unknown 3D workspace. In our future work, we will perform experiments utilizing real robots in order to more rigorously prove the proposed 3D foraging strategy.

Notably, each carrier agent is assigned to a puck and moves along the path to its designated puck in order to carry the puck. In practice, each puck may have distinct weights and distinct sizes. The maximum weight that can be carried by a carrier agent is limited. In addition, the path length from the base to each puck may be different. Considering these various aspects, it is possible to optimize the assignment of each carrier agent such that carrier agents can carry all of the pucks to the base within a minimum time interval. In the future, we intend to solve this optimal assignment problem.

## Data Availability

Data are available upon reasonable request.
